# Educational programmes for reducing depression, anxiety, and stress symptoms among migrant workers: a scoping review and evidence synthesis

**DOI:** 10.1186/s12889-025-25775-6

**Published:** 2025-12-02

**Authors:** Crystal Ying Chan, Harley Hiu-yung Kwok, Wai Tong Chien, Fish Pui-yu Ip, Danna Camille Vargas, Shannon Yuen, Benjamin Wong, Maggie Ying Yee Li, Becky Hoi, Joyce Ho Yi Chan, Eliza Lai-yi Wong

**Affiliations:** 1 Jockey Club School of Public Health and Primary Care, School of Public Health, Prince of Wales Hospital, Prince of Wales Hospital, The Chinese University of Hong Kong, Hong Kong, China; 2 Nethersole School of Nursing, Faculty of Medicine, The Chinese University of Hong Kong, Hong Kong, China; 3 International Domestic Workers Federation, Hong Kong, China

## Abstract

**Background:**

Migrant workers face additional stress from language and cultural barriers in host countries. Educational programs focus on supporting acculturation of migrant workers are often believed to help stress-relief by providing knowledge for their language learning, cultural understanding, and inclusive practises, but no conclusion has been made on the effectiveness. This review aims to address this gap by examining the impact of acculturation programmes in managing psychological distress among migrant workers, with anxiety and depression as primary outcomes, and stress as secondary outcome.

**Methods:**

We searched six bibliographic databases (Embase, Medline, CINAHL Complete, PsycInfo, SocINDEX, and Cochrane Library) from inception to 12 September 2024 using keywords “migrant workers AND education AND (stress OR anxiety OR depress)” and grey literatures.

**Results:**

Eleven articles were critically appraised as identified from 4795 searched titles. Included studies were primarily conducted in the United States, involved predominately blue-collar workers/ health care workers, and females. Seven types of interventions were identified. Specifically, positive impacts on stress levels were observed following lay health educators’ intervention (MD: -4.03, *p* < 0.01 in Latina migrants), peer groups, and spiritual retreat courses demonstrated significant evidence of reducing stress. Peer groups resulted in a significant improvement in perceived stress levels among resettled working refugees (MD: -5.00, *p* < 0.0001). Similarly, spiritual retreat courses significantly improved perceived stress levels in Nigerian women workers, with a mean difference of -4.05 (*p* < 0.01). However, improvement in anxiety after life skills trainings was insignificant. Overall, such programs can effectively address stress and depression, ultimately improving the overall well-being of migrant workers in host countries.

**Conclusions:**

Acculturation programmes covering life skills and work skills are common approaches in supporting migrant workers’ mental health. Overall, the findings suggest that migrant-local support groups, lay health educators’ interventions, peer groups, and spiritual retreat courses, can effectively address stress and depression among migrant workers. However, heterogeneity exists between studies. Acculturation programmes constitute feasible solutions to address the global mental health crisis. Future research should be done to investigate acculturative educational programmes impact in a larger population.

**Registration:**

This review was registered on PROSPERO (CRD42021292998).

**Supplementary Information:**

The online version contains supplementary material available at 10.1186/s12889-025-25775-6.

## Background

Migrant workers account for 167.7 millions employees globally, with over 50% working in industrialized economies [1]. Migrant workers as defined by the International Organization for Migration (IOM) as individuals engaged in remunerated activity outside their country of origin, typically without permanent residency rights or pathways to citizenship. Migrant workers from low- and middle-income countries are more likely to be exploited and are vulnerable to poor psychological health, exacerbated by cultural and language barriers, financial burden, limited social support networks, and inadequate social protection in their host countries [2, 3]. Moving to a new territory with differing customs and cultural practices, can exert substantial burdens on migrants [4–6]. Adverse environments, limited knowledge on employment laws, exploitation due to poor working conditions, and language barriers in the host countries, exacerbated a sense of powerlessness among workers, hence increasing their susceptibility to mental health issues [7–10]. Despite extensive research on psychosocial interventions for displaced persons, notable gaps persist in the evidence to identify effective interventions for common mental health problems such as depression, anxiety, and stress symptoms [11, 12]. This highlights the need for a systematic synthesis that examines educational programmes aimed at reducing these symptoms among migrant workers, in order to guide future interventions and policy for these frequently underserved populations.

Acculturation is believed to be significantly impactful on the mental health of migrant workers through adopting their values, behaviours, customs, language, and identifies in a different cultural environment [13, 14]. Substantial contemporary evidence demonstrates that acculturation, which is understood as the multidimensional process through which individuals navigate cultural adaptation when residing in a new cultural context, remains a critical determinant of mental health outcomes, including amongst temporary migrant workers, and warrants systematic examination [15–17].​ Crucially, empirical research consistently demonstrates that integration, not assimilation, is associated with the most favourable mental health outcomes [18], whilst marginalisation and separation are linked to elevated psychological distress [19]. Understanding acculturative stress (the psychological strain arising from navigating cultural transitions, language barriers, discrimination, economic precarity, and systemic marginalisation) is essential for identifying modifiable risk factors and designing culturally responsive mental health interventions [15].​ Skills in adapting to new contexts are essential for cross-cultural adjustments, and can protect migrant workers from stress, anxiety and depression [20–24]. Contrasting with the challenges experienced by low-skilled migrant workers, skilled individuals possess more bargaining power and thus are less vulnerable to exploitation as well as more resilient to adverse mental health [7, 25]. Thus, having access to the necessary knowledge and skills to adeptly navigate daily life and employment can protect migrant workers from preventable stressors in their workplaces. By empowering migrant workers with such knowledge and skills, these interventions help migrant workers to develop their capacity, build their resilience against life and work adversities, protect them from stress, and facilitate the acculturation process in their host countries. However, these efforts must not frame acculturation as a normative mental health solution, nor overlook systemic drivers of poor well-being such as labor exploitation or discrimination.

Given the breadth and diverse nature of literature, scoping review is used in this study to identify existing evidence gaps and determine whether a full systematic review is warranted in the future [26, 27]. This review aims to identify and synthesise the evidence of existing acculturative educational interventions targeted at the prevention, management, or deterrence of the development or recurrence of stress of migrant workers in a global context. Studies solely on refugees were excluded. In accordance with the PROSPERO registration (CRD42021292998), the primary outcomes of this review were *anxiety*, *depression*, and *diagnoses of anxiety and depression*. These outcomes were selected based on their central relevance to the study question and their consistent measurement across eligible studies. During the review process, stress was added as an additional secondary outcome to enhance the comprehensiveness of the analysis and capture broader indicators of mental health. This decision was made prior to data analysis and has been transparently reported here to ensure adherence to best practices in protocol amendment disclosure. All outcomes were analysed quantitatively where possible, using standardised mean differences or odds ratios, depending on data type and availability, with corresponding 95% confidence intervals.

We focused on the knowledge/information dissemination of the educational interventions with clear course structure and designated education outputs in their design. Regular schooling in host countries was excluded. The interventions should provide education in at least one of these aspects: (1) work skills, (2) life skills, and (3) both life-and-work skills and psychological care.

## Methods

### Search strategies

This review was conducted and reported in accordance with Levac, Colquhoun and O’Brien [27], and the Preferred Reporting Items for Systematic Reviews and Meta-Analyses extension for Scoping Reviews (PRISMA-ScR) guidelines [28]. In order to include most of the relevant articles, we used the a more lenient combination of keywords “migrant workers”, “education”, “depression”, “anxiety” and “stress” to conduct searches on 11 December 2021, and updated on 16 October 2025 in six bibliographic databases (including Embase (1910-Present), Medline (1946-Present), CINAHL (1941-Present), APA PsycInfo (1806-Present), SocINDEX (1895-Present), and Cochrane Library (1996-Present), and nine sources of grey literature/search engines (including International Labour Organisation, the International Organisation of Migrant, the World Health Organisation, the United Nations Refugee Agency, the UN-Habitat, the European Commission, the United Nations Educational, Scientific and Cultural Organisation, and the United Nations Development Programme, Google, Google Scholar, and Scopus). Reference lists and an additional list of titles suggested by our LEAs were checked. We screened both quantitative and qualitative studies published in peer-reviewed journal and grey literature databases, to optimise data coverage from the existing literature. Upon retrieval of a grey literature, there was a discussion between the two senior authors (CYC, ELYW) to assess the study design and use corresponding risk of bias tool to assess the quality of the study. Studies were eligible for inclusion irrespective of study design, encompassing both quantitative and qualitative methodologies, as long as extractable data were available on the psychological conditions of migrant workers before, during, or after their participation in educational interventions. The study population comprised international migrant workers of any age, gender, race, nationality, or geographic region, who were engaged in paid employment in host countries. Studies that included both migrant and non-migrant participants were retained only when data could be separately extracted for migrants. To ensure focus on mental health outcomes, only findings related to anxiety, depression, and stress were extracted when broader psychological variables were reported. Eligible educational interventions included structured programmes aimed at enhancing participants’ knowledge or skills relevant to their work or daily living—in particular, (1) work-related skills training (e.g. vocational or professional development), (2) life skills education (e.g. language, health literacy, or social adjustment training), and (3) combined work-and-life skill or psychoeducational interventions focusing on well-being, resilience, or mental health literacy. Programmes were required to report defined learning objectives or deliverables and include information dissemination components such as classes, workshops, or educational materials. Interventions limited to regular formal schooling were excluded. Studies written in English or Chinese and available in full text, including peer-reviewed articles and grey literature (such as reports), were considered eligible. Publications without accessible full texts were excluded from the review. We excluded articles (1) where no full text was found, (2) were not written in English or Chinese, (3) solely on refugees. In this review, we have adopted the PICO structure (population: migrant worker, intervention: education or empowerment or life skill, comparison group: N/A, outcome: stress or anxiety* or depression*) statement in formulating the search strategies in different databases. Full search terms for each databases are detailed in Additional files 1 & 2.

### Data extraction and analysis

All searched titles were imported into Covidence.org [29] for de-duplication, double screening, and double extraction in all title/abstract and full-text screening, by two independent reviewers with epidemiology or psychology training. Quality were assessed using the Joanna Briggs Institute (JBI) Critical Appraisal Tools [30] and the Mixed Methods Appraisal Tool (MMTA) Version 2018 since they are suitable for various kind of study type [31]. For randomised controlled trials (RCTs), the Cochrane Risk of Bias Tool (RoB 2.0) was also applied as triangulation in accordance to our original protocol [32]. Evidence synthesis was done by critically appraising the included articles’ contexts, populations, outcome indicators, and effect sizes in terms of the levels of evidence and clarity. Effect sizes with statistical significance of interventions implemented in each of the included studies were compared. Senior authors further determined whether the interventions had positive, negative, or obscure impacts, and summarised the results through generalised phrases.

### Involvement of lived experience advisors (LEAs)

No patient was involved in this secondary data analysis. However, we involved LEAs with different genders and nationalities formed a consultation panel to provide insights in idea formulation, data screening and interpretation of the study results. We purposively recruited advisors from seven non-profit organisations (NPOs) serving migrant worker in Hong Kong, Malaysia, Philippines, Japan, and Taiwan. Our advisors were (1) working or worked outside their home countries; and (2) leading an NPO serving migrant workers at the time of recruitment. Detailed account of advisor involvement can be found in Additional file 3.

### Ethical considerations

Ethical approval was not required as we relied on published data.

## Results

### Studies description

**Fig. 1:**
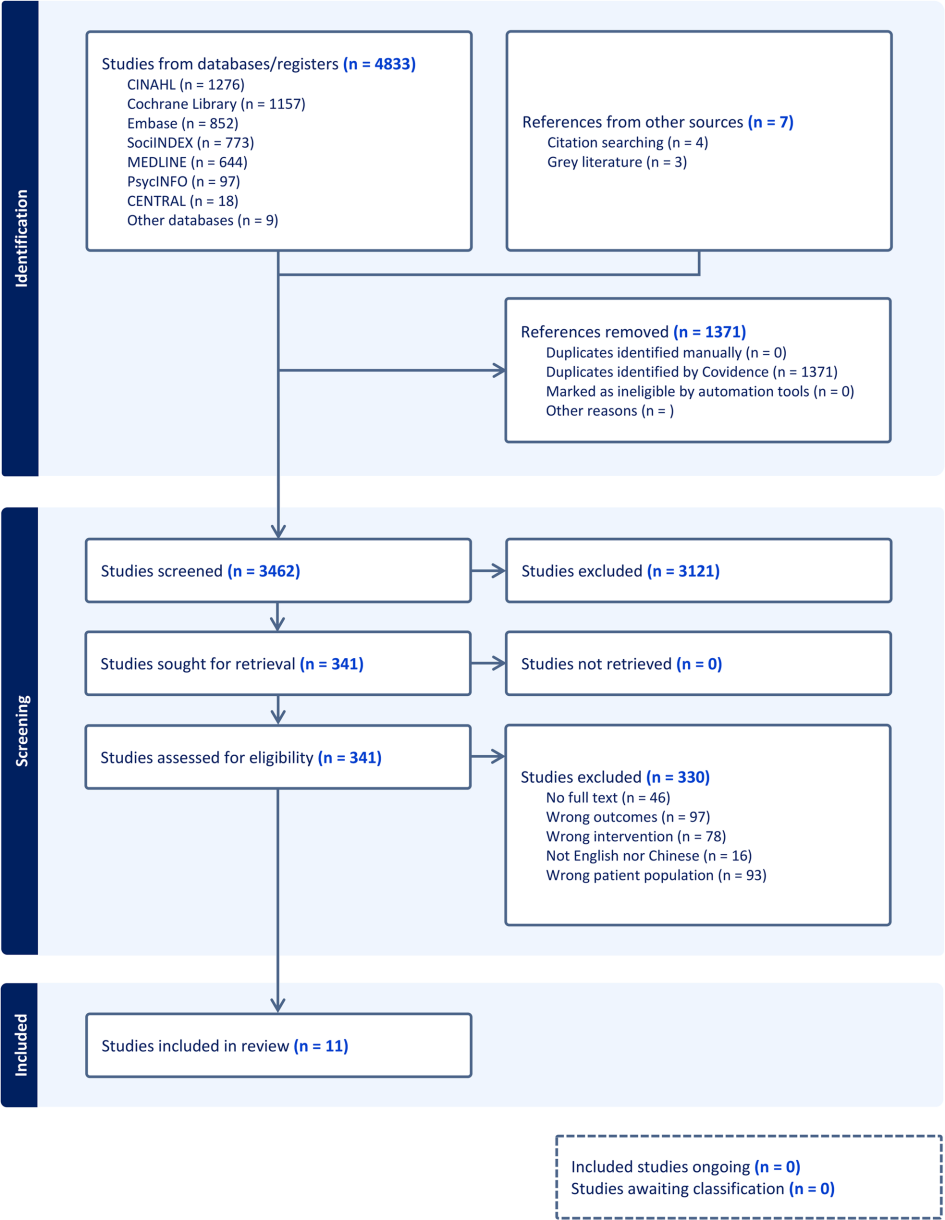
PRISMA-ScR Flowchart

Of the 4795 titles identified, 11 were included after application of inclusion and exclusion criteria of mental health intervention on migrant workers (see Fig. 1). We have included studies reporting quantitative outcomes, qualitative outcomes, and both. A total of nine studies reported quantitative outcomes, in which four studies focus on work skill interventions (see Table 1); and five on life skill interventions (see Table 2). Another five studies reported qualitative narratives, in which two studies on work skill interventions (see Table 3), and three on life skill interventions (see Table 4). A majority of included studies were conducted in the United States (*n* = 6) [33–38]. There were three from the European Union countries (one from Netherlands [39], one from Denmark [40] and one from Germany [41]) and two from Taiwan [42, 43].

**Table 1:** Summary of quantitative content in work skills from the included studies (*n* = 4)

Study (publication year) (*n*)	Country	Study design	Population/Settings/Recruitment	Intervention design	Type	Outcome: Stress	Outcome: Anxiety	Outcome: Depression	Quality assessment
Design #1 Work skills: In-job training									
Wu (2005) (*n* = 35)	Taiwan	Quasi-experimental design	35 migrant nurse aids working **≥** 6 h per day in 10 long-term care facilities in Taiwan (16 intervention; 19 control). Purposive sampling from 10 long-term care facilities in the Shihlin and Peitou areas of Taipei	Intervention: In-service occupational training program - Implemented for foreign nurse aides on physical assessment for residents, nurse care for common diseases, emergency techniques, wound care, rehabilitative exercises, nutrition for the elderly, stress management, communicable disease protection for facilities, occupational risk protection, and physical exercise - Audience: Migrant workers - Type: Group-based intervention - Intervention length: 3 months	Group-based	Stress level^1^ was assessed by the following regards: General job tasks: At baseline: 12.50; after intervention: (Z=−0.44, *p* = 0.66) Patient care tasks: At baseline: 14.29; after intervention: (Z = 0.86, *p* = 0.39) Relationship with co-workers: At baseline: 8.13; after intervention: (Z = 0.15, *p* = 0.88) Relationship with supervisors: At baseline: 10.47; after intervention: (Z=−0.013, *p* = 0.90) Workload/scheduling: At baseline: 11.13; after intervention: (Z = 2.01, *p* < 0.05) Facilities design/maintenance: At baseline: 10.20; after intervention: (Z = 1.38, *p* = 0.17)			High (9 out of 9) Quality rating*: B
Chuang (2018) (*n* = 85)	Taiwan	Cross-sectional study	85 home-based female migrant care workers aged under 60 working for patients at Chang Gung Memorial Hospital, Kaohsiung, Taiwan. Convenient sampling at working hospital	Intervention: Occupational training in host country - Includes training on basic nursing, communication and emergency management skills, mental health and stress adaptation, the use of proper body mechanics techniques, use of assistive devices and etc. - Consists of 50–60 h of lecture, and 40 h of clinical practice. - Audience: Migrant workers - Type: Group-based intervention - Intervention length: 90 h (50–60 h of lectures and 40 h of clinical practice)	Group-based	Avoidance of additional stress^2^ with odds ratio 5.48 (*p* < 0.05)			High (5 out of 7) Quality rating*: D
Design #2: Work skills: Multi-level workplace engagement intervention									
Smith (2013) (*n* = 14)	Denmark	Mixed-methods study (Individual semi-structured interview & survey)	14 cleaners employed at the school (85.7% were migrants). Voluntary basis recruitment	Intervention: Multi-component, psychosocial work environment in a multi-ethnics Danish cleaning workplace, involving work-related language lessons and vocational training courses for migrant workers, and workshop on job satisfaction and teamwork, more frequent staff meetings, and additional social events for workers with different ethnicities - Audience: Migrant workers, non-migrants in the same community - Type: Individual- and group-based intervention - Intervention length: 8 months	Group- and individual-based	No significant effect on improving either one of the outcomes of interest^3^			High (13 out of 15) Quality rating*: B
Busch (2017) (*n* = 421)	Germany	Mixed-methods study (semi-structured interviews and observation)	421 low-skilled workers (50% were immigrants) [252 intervention, 169 control] working in a welfare facility, a service production company, and a car accessory manufacturing plant. Voluntary basis recruitment	Intervention: Organisational health intervention for low-skilled workers and their managers - Given to low-skilled workers (through peer mentoring and 3 training of 4 h each), line manager (through 4 training sessions of 4 h each), and the company’s senior and middle managers (through workshops on management concepts) - Audience: migrant worker, non-migrants in the same community, and employers - Type: Individual- and group-based intervention - Intervention length: 10 weeks	Group- and individual-based	Mean stress score^4^ at post-intervention was 0.054 (SD = 0.044, *p* > 0.05, exact p-value not reported) lower than that at baseline; mean stress score after follow-up period was 0.028 (SD = 0.044, *p* > 0.05, exact p-value not reported) lower than that at baseline			High (11 out of 14) Quality rating*: B

Remarks for outcome measures used: 1 Measured by the modified Work Stressor Inventory (WSI); 2 Measured by the 13-item Caregiver Strain Index (CSI); 3 Measured by Copenhagen Psychosocial Questionnaire (COPSOQ); 4 Measured by a simplified 9-item scale by Mohr and Müller

*Quality Rating Scheme for Studies and Other Evidence with rating A-E. A: Properly powered and conducted randomised clinical trial; systematic review with meta-analysis; B: Well-designed controlled trial without randomisation; prospective comparative cohort trial; C: Case-control studies; retrospective cohort study; D: Case series with or without intervention; cross-sectional study; E: Opinion of respected authorities; case reports

**Table 2:** Summary of quantitative content in life skills from the included studies (*n* = 5)

Study (publication year) (*n*)	Country	Study design	Population/Settings/Recruitment	Intervention design		Type	Outcome: Stress	Outcome: Anxiety	Outcome: Depression	Quality assessment
Design #3: Life skills: Lay health educator empowerment										
Tran (2014) (*n* = 58)	United States	Quasi-experimental design	58 Latina migrant women aged 18 years old or above (58.6% were workers) Recruited by promotoras	Intervention: Lay health educator intervention that recruited and trained migrant peers to reach out and support migrant workers for health resources sharing, and mental support - Recruited and trained Latina migrant “Promotoras” to reach out and support other Latina migrant women through health resources sharing, information exchange and mental support - Audience: Migrant workers, non-workers in the migrant community - Type: Individual based intervention - Intervention length: did not specify	Individual-based		Mean stress score^7^ decreased from 26.68 (SD = 4.32) to 22.65 (*p* < 0.01) right after the intervention		Mean depression score^8^ decreased from 16.45 (SD = 17.14) to 8.47 (*p* = 0.01) right after the intervention	Medium (6 out of 8) Quality rating*: B
Design #4: Life skills: Patient education										
Kocken (2008) (*n* = 139)	The Netherlands	Randomised controlled trial	139 female Turkish and Moroccan migrant patients with complain of stress-related symptoms (14% were worker). Clinic patients recruited on a voluntary basis	Intervention: Information transfer group and one-to-one counselling intervention on how to seek mental healthcare - Facilitates the communication between the general practitioner and the participant - Audience: Migrant workers - Type: Individual- and group-based intervention - Intervention length: Mean intervention duration per patient was 12 months	Group- and individual-based		Mean stress score^9^ decreased from 5.41 (SD = 2.98) to 4.82 (SD = 2.97, *p* = 0.34) right after the intervention			Medium (8 out of 13 using JBI checklist; LOW risk of bias using Cochrane ROB2) Quality rating*: A
Design #5: Life skills: Peer support groups										
Le (2021) (*n* = 86)	United States	Mixed-methods study	86 Latina immigrant women with depressive features who sought food and nutrition support (% of worker was unknown; mean personal income USD 886.7 [SD = 467.8]). Voluntary basis recruitment	Intervention: Educational intervention on stress management for pregnant women, including balancing parenting and work - Empowered women about the relationship between mood and: (a) thoughts, (b) activities, and (c) relationships with others, consistent with a cognitive-behavioural and attachment theory and adapted to the contextual factors of low-income Latinas - Audience: Migrant workers - Type: Group-based - Intervention length: 6 weeks	Group-based				Mean depression score^10^ decreased from 18.66 (SD = 5.36; *p* < 0.001) to 14.71 (SD = 6.68; *p* = 0.001) right after the intervention; later decreased to 13.54 (SD = 4.10; *p* < 0.001) 3 months after completion of intervention	High (13 out of 14) Quality rating*: B
Design #6: Life skills: Psychoeducation counselling										
Hovey (2014) (*n* = 6)	United States	Cohort study	6 female migrant farmworkers in the Montrose Area of Western Colorado. Voluntary basis recruitment	Intervention: Psychoeducation and support group discussion - Culturally adapted, group-based cognitive-behavioural therapy - Topics include: understanding and coping against stress, anxiety and depression; increasing hopefulness and self-esteem; family issues related to nurturance and childrearing, discipline, family functioning and communication, and acculturation gaps (i.e., children acculturating at a quicker pace than parents); and understanding and recovering from domestic violence and other traumatic experiences - Audience: Migrant workers - Type: Group based intervention - Intervention length: 6 sessions; once per week	Group-based		Mean stress score^13^ decreased from 80 (SD = 25.6) to 64.5 (SD = 21.4, *p* = 0.01) right after the intervention; later decreased to 58.5 (SD = 18.1, *p* = 0.058) after post-intervention follow-up period	Mean anxiety score^14^ decreased from 75.3 (SD = 19.1) to 65 (SD = 6.4, *p* = 0.13) after post-intervention follow-up period	Mean depression score^8^ decreased from 34.4 (SD = 14.1) to 16.6 (SD = 8,*p* = 0.02) right after the intervention; later decreased to 15.5 (SD = 4.7, *p* = 0.02) after post-intervention follow-up period	Low (3 out of 7) Quality rating*: B
Design #7: Life skills: Spiritual retreat course										
Ekwonye (2018) (*n* = 88)	United States	Non-randomised experimental study	88 Nigerian migrant women with religious in United States (100% were worker). Convenience sampling from church	Intervention: Spiritual-based retreat activities involving communal singing, meditation, and open dialogue conversation with a retreat moderator - Led by a retreat coordinator (a clergy) from the church, involving a communal singing of the Divine office, followed by a 30-minute mindfulness meditation and a Mass in the morning. Then, participants had an hour of open dialogue with the moderator twice a day. - Provided them with renewed energy, inner resources to discern what truly matters in life, enhanced sense of meaning and purpose, and improved self-efficacy - Audience: Migrant workers - Type: Group-based intervention - intervention length: 6 days	Group-based				Mean stress score^11^ decreased from 19.51 to 13.16 right after the intervention (*p* < 0.01)	High (7 out of 8) Quality rating*: B

Remarks for outcome measures used: 5 Measured by the modified versions of the Distress and Happiness Sub-scales of Rumbaut’s Psychological Well-being Scale; 6 Measured by Satisfaction with Life Areas (SLA) scale; 7 Measured by the 14-item Perceived Stress Scale; 8 Measured by the 20-item Center for Epidemiologic Studies Depression Scale (CES-D); 9 Measured by the 90-items symptom checklist (SCL-90); 10 Measured by Postpartum Depression Screening Scale, Short-form (PDSS-SF); 11 Measured by Measured by the 10-item Perceived Stress Scale; 12 Measured by the Hopkins Symptom Checklist-25 (HSCL-25); 13 Measured by the 39-item Migrant Farmworker Stress Inventory (MFWSI); 14 Measured by the 24-item Personality Assessment Inventory (PAI), Anxiety Scale

*Quality Rating Scheme for Studies and Other Evidence with rating A-E. A: Properly powered and conducted randomised clinical trial; systematic review with meta-analysis; B: Well-designed controlled trial without randomisation; prospective comparative cohort trial; C: Case-control studies; retrospective cohort study; D: Case series with or without intervention; cross-sectional study; E: Opinion of respected authorities; case reports

**Table 3:** Summary of qualitative content in work skills from the included studies (*n* = 2)

Study (publication year) (*n*)	Country	Study design	Population/Settings/Recruitment	Intervention design	Type	Outcome: Stress	Outcome: Anxiety	Outcome: Depression	Quality assessment
Design #2: Work skills: Multi-level workplace engagement intervention									
Smith (2013)	Denmark	Mixed-methods study (Grounded theory)	Please refer to Table 1.			Stress (as a component in psychosocial work environment)			High (13 out of 15) Quality rating*: B
						1. Improved communication and collaboration within the group of cleaners			
						2. Improved trust and sense of belonging among new-comer cleaners			
						3. Improved communication between cleaners and the supervisor through improved cleaners’ language proficiency			
Busch (2017)	Germany	Mixed-methods study	Please refer to Table 1.			1. Reduced stress through improving peer support among through training			High (11 out of 14) Quality rating*: B
						2. Managers are not motivated to participate and provide support			
						Five Mechanisms of Change derived:			
						1. Company management encouragement mechanism (Company managers were encouraged to participate because of the target-group specific interventions at low costs)			
						2. Role model mechanism (Peer mentors were highly motivated to participate and to fulfill their new roles)			
						3. Peer-mentor support mechanism (peer-mentors increasingly supported their coworkers)			
						4. Participative work improvement mechanism (Peer-mentors initiated high-quality changes at work in a participative way such as improving the organization of shift work, creating a new rest area, cleaning up workplaces and reducing social conflicts at work)			
						5. Line manager support mechanism (Support given by line managers during the implementation process)			

*Quality Rating Scheme for Studies and Other Evidence with rating A-E. A: Properly powered and conducted randomised clinical trial; systematic review with meta-analysis; B: Well-designed controlled trial without randomisation; prospective comparative cohort trial; C: Case-control studies; retrospective cohort study; D: Case series with or without intervention; cross-sectional study; E: Opinion of respected authorities; case reports

**Table 4:** Summary of qualitative content in life skills from the included studies (*n* = 3)

Study (publication year) (*n*)	Country	Study design	Population/Settings/ Recruitment	Intervention design	Type	Outcome: Stress	Outcome: Anxiety	Outcome: Depression	Quality assessment
Design #5: Life skills: Peer groups intervention									
Le (2021)	United States	Mixed-methods study (Thematic content analysis, Concordance analysis)	Please refer to Table 1.			Stress (Psychological well-being) and 1. Increase emotional awareness: awareness of mood changes (learnt skill) 2. Learn to apply cognitive behavioral concepts: behavioural activation and cognitive re-structuring 3. Value the group experience: social comparison and social support		Depression (symptoms) Improved intervention outcome: decrease in depressive symptoms	High (13 out of 14) Quality rating*: B
Design #6: Life skills: Psychoeducation counselling									
Weiss (2011)	United States	Case series	2 migrants with social anxiety disorder recruited from a mental clinic in the United States	Intervention: Psychoeducation intervention - A part of a cognitive-behavioral therapy (CBT) for the treatment of Social Anxiety Disorder (SAD) - Audience: Migrant workers - Type: Individual-based intervention - Intervention length: Mr. M: 17 sessions Ling: 16 sessions	Individual-based		Anxiety (Social Anxiety Disorder) CBT for SAD in two ethnic minority clients were successful of clients with different backgrounds, differing time in the United States, and differing proficiency with English		Medium (5 out of 9) Quality rating*: B
Mitschke (2017)	United States	Qualitative research (Individual semi-structured interviews) (Thematic analysis)	30 resettled refugees from Burundi, Burma, Congo, Rwanda, and Bhutan (10.7% were worker). Voluntary basis recruitment	Intervention: Psycho-educational group meetings, office-based counseling, and home-based counseling - Community-based - Audience: Migrant workers, adult refugees - Type: Individual- and group-based intervention - Intervention length: 8 weeks	Group- and individual-based		Anxiety and depression (Symptoms of anxiety and depression) Group Program Structure: 1. Social Support (participating in the mental health sup-port group with their peers helped to reduce feelings of social isolation) 2. Mutual Aid (the assertion that the group program structure was an effective mechanism to create a sense of shared responsibility and obligation to one’s fellow community members, the mental health group was an opportunity to share ideas and knowledge and also a place to receive and exchange practical help from others) 3. Empowerment (participants sense power through group membership) Program Content: 1. System Navigation (participants expressed the need for assistance in navigating the health and social services landscape in the US, especially skills and knowledge related to accessing healt^1^h and medical services) 2. Literacy and Language (participants express the need for English language literacy assistance, and assistance in attaining US Citizenship) 3. Advocacy (the need for advocacy on behalf of the participants, especially related to interactions with employers and medical or social service settings) 4. Counseling (the importance of building the relationship between the individual client and the counselor as an essential component of establishing trust and honesty in a mental health intervention) 5. Ethnic-specific Differences (differences between the interests demonstrated by the interviewees)		Medium (6 out of 10) Quality rating*: D

*Quality Rating Scheme for Studies and Other Evidence with rating A-E. A: Properly powered and conducted randomised clinical trial; systematic review with meta-analysis; B: Well-designed controlled trial without randomisation; prospective comparative cohort trial; C: Case-control studies; retrospective cohort study; D: Case series with or without intervention; cross-sectional study; E: Opinion of respected authorities; case report

The mean age of the included participants ranged from 29 [43] to 51 [36]. Half of the studies involved predominantly or only female participants [33, 35, 37, 39, 42, 44]. The migrant population reviewed came from a wide range of socio-economic backgrounds, including patients with stress, anxiety or depressive features [35, 38, 39], women [33, 37], blue-collar workers (e.g., farmworkers, cleaners etc.) [40, 41, 44], and care workers (e.g., nurses and home care workers) [42, 43].

### Quality assessment

All 12 studies had adequate reporting for quality assessment (see Additional file 4). Five articles were ranked as high quality [33, 35, 40, 41, 43], five as medium quality [36–39, 42], and one as low quality [44]. Study rated as low quality was interpreted with caution during analysis, but not excluded.

### Intervention characteristics

We classified included articles into work skill (focuses on skills facilitating job seeking and work activities) and life skill educational interventions (focuses on skills facilitating cultural adaption in host countries). Additional file 5 summarises the skill enriched through education programs identified in the review.

We identified two types of work skill educations: 1) in-job trainings [42, 43] aims to improve clinical care skills of new migrant healthcare workers; and 2)multi-level workplace engagement intervention [40, 41] that arises through an organisational effort to introduce work-related language lessons and vocational training courses for migrant workers. One example of the in-job trainings included in-service occupational training for migrant nurse aids in Taiwan, which focused on the skills required in physical assessments, wound care, and rehabilitative care skills for patients living in long-term care facilities, as documented by Wu and colleagues [43]. A mixed-method study captured a multi-level workplace engagement intervention in a multi-ethnicity Danish cleaning workplace, which aimed to improve the workers’ competence by introducing work-related language lessons and vocational training courses for migrant workers [40]. It hoped to improve their job satisfaction and facilitate team work through increasing the frequency of staff meetings, providing educational workshops and creating additional social events among workers over an eight-month intervention period.

Alternatively, life skill education was delivered in the form of (1) lay health educator empowerment [37]; (2) patient education [39]; (3) peer support groups intervention [35], (4) psychoeducation counselling [36, 38, 44], or 6) spiritual retreat course [33]. All the life skill educational interventions took place in the community where the workers were living, instead of their workplaces. Individual-based interventions included lay health educator empowerment interventions that recruited and trained migrant peers to reach out and support migrant workers [37], and psychoeducation provided to migrant workers with social anxiety [38]. Group-based interventions consisting of migrant peers only [35, 45]were identified in this review. In the US, a spiritual retreat course which contained group-based meditation and open dialogue conversation led by a professional moderator, was organised for Catholic Nigerian workers to relieve their stress [33].

We identified wide range of life-and-work skills in this review. Work skills that were identified from the review included emotional and instrumental support skills [41], skills to hold meetings effectively [41], incorporate worker support elements in daily management [41], improving work environment through participatory advocacy [36, 41, 46, 47], vocational training [40, 42, 43], and job application training [36, 40, 47]. Among them, one study [41] provided work skills trainings to both the manager and the migrant workers. We also identified targeted interventions to support life skills, including techniques such as host country language [36, 40], stress management [33, 35–39, 43], knowledge to access health and welfare resources in the host countries [36, 37, 39], social networking and peer knowledge exchange [36, 37, 43], health knowledge on mental illnesses [37, 39], and skills in parenting and handling family conflicts [44]. All the education focusing on life skills was targeted at migrant workers only.

### Intervention effect

#### Quantitative analysis

Tables 1 and 2 summarises intervention impacts on the migrant workers’ stress (*n* = 7), anxiety (*n* = 1), and depression (*n* = 2). Effect sizes of the outcome measurements were recorded to demonstrate the significance of change in the intervention outcomes, despite the outcome measurements being non-standardised across studies.

Life skill educations, instead of work skill educations, were found to be beneficial to stress and depressive symptoms in migrant workers in medium to high quality evidence. Specifically, lay health educator empowerment (effect size = −4.03, *p* < 0.01 in a medium quality quasi-experimental study in US [37]), and spiritual retreat courses (effect size = −4.05, *p* < 0.01 in a high quality non-randomised experimental study in US [33]) showed significant positive impacts on the workers’ stress level. Additionally, two studies found significant improvement in migrant workers’ depression symptoms post-intervention, including a high quality mixed method study on pregnant women joining peer support groups (effect size = −3.95, *p* = 0.001 [35]), and a low quality cohort study documenting effect of a psychoeducation counselling for Latina farmworkers (effect size = −17.8, *p* = 0.02 [44]).

Few studies quantitatively evaluated intervention impacts on anxiety. Two studies evaluated the impacts of life skill education on anxiety levels but results were conflicting [44]. There was no quantitative investigation done on the impacts of work skill education on migrant workers’ anxiety and depression levels.

#### Qualitative descriptions

Four studies included qualitative description of intervention effect on stress [40, 41] (*n* = 2), anxiety [36, 38] (*n* = 2), or depression [35, 36] (*n* = 2) of migrant workers, of which three were part of a mixed-method study to explore the mechanism of intervention impacts on mental health outcomes. Two high-quality studies conducted in the US found that life skill educations, such as peer groups, could provide stress-relief through teaching new skills on emotional awareness and cognitive behavioural intervention [35]; while another two high quality studies which took place in Denmark [40] and Germany [41], described how multi-level workplace engagement intervention, which involves both employers and employees, could relieve stress through improving communication and collaboration within groups of cleaners [40, 41] and between cleaners and their manager [40]. Life skill educations were also reported to be beneficial to workers with anxiety [36, 38] and depression symptoms in qualitative studies [35, 36].

### LEA panel discussion

LEAs concluded that the below features of educational interventions could be important for building social/peer support for migrants, including: (1) adopting culturally-sensitive approaches (including spiritual-training and lay health educator empowerment) in health promotion programmes; (2) providing affordable professional support for migrant workers with clinical mental health needs; and (3) needing sustainable manpower and financial support for NPOs and community agencies, to provide support interventions for improving the migrant workers’ mental health. Detailed information of these engagements was elaborated in Additional file 6.

## Discussion

### Significance of this study

This is the first scoping review consolidating the characteristics and impacts of acculturative educational interventions on migrant workers’ anxiety and depression symptoms, with stress as secondary outcomes. Despite the scarcity of literature, we identified moderate to high quality evidence supporting the beneficial impacts of life skill education on stress, anxiety and depression management in this population, through enhancing communication within workers’ groups. By contrast, work skill interventions, such as in-job training and multi-level workplace engagement interventions, had no significant impact on the migrant workers’ stress level. This observation aligns with our previous understandings on the interlinked relationship between social isolation [48–50], coping strategies [51, 52], adaptation to new lives in host countries [53–55], and the mental wellbeing of the worker population. In lieu of a therapeutic option, our research demonstrates that life skills educational interventions are a feasible and promising solution to address the mental health crisis suffered by the migrant worker population, with evidence of effectiveness available from populations across different nations, cultures, ethnicities, and occupations.

Our findings illuminate a new path to address the mental health crisis of global migrant workers, from an educational and empowerment perspective. Acculturative stress, which can occur when migrant workers have to adapt to the new socio-economic culture of the host countries [56, 57], is associated with increased risk of suffering adverse mental health outcomes including psychological stress, anxiety and depression [58–60]. While migrants can experience different stresses during acculturation, mastering life skill including language learning and expanding their social circles in their host country are crucial for helping them to adapt to their new environment, enabling them to manage complex situations and identify the resources they require to satisfy their needs [61, 62]. Previous systematic reviews [5] have concluded that migrant workers can respond to acculturative stress by employing specific coping strategies, including emotion-focused (e.g., seeking emotional support), problem-focused (e.g., learning and seeking help), and appraisal-focused strategies (e.g., being hopeful and humorous). Our study identified practical examples of life skill training interventions that could help migrant workers develop coping strategies through a series of individual or group-based activities. For instance, through providing host country language training to improve communication between migrant workers and their local peers [36, 40, 46, 47], and training on stress management skills to strengthen workers’ abilities to regulate their mood during their early days after migrating [33, 35–39, 43, 45, 46].

Another possible pathway explaining the stress-relieving effect of life skill education, might be the community support network built during the intervention, which is well-known to relieve stress, anxiety and depressive symptoms among migrant workers [23, 63, 64]. Empowering and strengthening peer support were also important aspects of life skill educations identified in this study; for example, lay health educator empowerment, and peer support groups interventions. Besides acquiring social networking skills to establish peer support through the educational sessions [36, 37, 43, 45–47], some life skill educational interventions involved trainings to empower workers to undertake advocacy to improve their work environments in a participative way [36, 46, 47]. Building workers’ capacity to understand their rights and to “voice” their needs through taking language classes and other life skill trainings, would empower them and improve their wellbeing through positive social exchange and engagement in the workplace [7]. Our study has identified practical examples of individual- and group-based interventions across various cultures and populations, which provide a scalable option for resilience building in the migrant population.

### Service recommendations

Despite the scarcity of literature, this study shed light on potential service options for improving migrant workers’ mental health. In terms of stress prevention and alleviation for this population, culturally appropriate life skills trainings are important strategies to adopt. Empowering cultural-sensitive media such as the lay health educator empowerment interventions with the same ethnicity as the migrants, can help reach out to marginalized populations. Group-based interventions, including support groups providing daily living skills exchange and spiritual retreat courses, can help relieve stress through encouraging social interaction. Likewise, we suggest organising group-based life skill training, which comprise the elements of promoting problem-solving skills alongside healthy lifestyle activities, to tackle anxiety and depression among migrant workers. Indeed, for migrant workers who wish to alleviate stress and cope with depressive symptoms, culturally-adopted and group-based psychoeducation can prove helpful over several sessions.

### Limitations

One of the limitations of our study is that the majority of the results was identified in the US, underrepresenting the European (23.9%) and the Arabic states’ migrant worker populations (13.9%) [1]. Due to the small sample size, the generalisability of the results of our included experimental studies is reduced (only three studies with *N* >100). Heterogeneity of study design, population, and outcome measures of the included study makes it not possible to conduct meta-analysis. In our study, we assessed the intervention’s impact on depression, anxiety and stress level by grouping them into life and work skill interventions, as well as subgroups such as “in-job training” and “multi-level workplace engagement intervention”. However, the impact of these interventions did not consider the variation of participation ethnicity, outcome measurement, and study design. Of these interventions did not consider the variation of participation ethnicity, outcome measurement, and study design.

Current research gap is that there was a lack of high-quality evidence from the included studies. More experimental studies such as randomised controlled trial and quasi-experimental trial should be implemented. In addition, cost-effectiveness analysis of different life-and-work educational interventions could be conducted, and therefore could provide a chance to quantify health utility in the migrant worker population. In terms of implementation, feasibility and adaptability studies of different types of life-and-work educations in migrant workers populations of different ethnicities and at various geographical locations, could be conducted. Future studies could involve participants coming from a diverse migrant worker population and in different workplace settings. Future studies should also be done to investigate life-and-skills education impact in a larger population. Study results could then shed light on the development of universal strategies for promoting life-and-work education in migrant communities globally.

## Conclusion

In sum, acculturative educational intervention is feasible and beneficial to in migrant workers’ mental health. Particular interventions such as migrant-local support groups, lay health educators’ interventions, peer groups, and spiritual retreat courses show evidence in addressing stress and depression risk among migrant workers. There is also a need for more consolidated efforts in engaging migrants’ workers in corporate governance, policy-making process, and community development through legal enforcement and community-wide migrant worker friendly campaigns. The continuation to conduct high-quality studies to investigate the cost-effectiveness of life-and-work educations in migrant populations with different backgrounds and in different geographic locations, including the Arabic countries, can provide further insights to the collective effort of improving migrant workers’ mental health globally.

## Supplementary Information

Supplementary Material 1. Additional file 1. List of databases searched and the search strategies used: listed full search term had been used. Additional file 2. Study inclusion criteria: outlined the study selection criteria. Additional file 3. Involvement of lived-experience advisors: detailed account of advisor involvement. Additional file 4. Quality assessment reports: quality assessment reports for all 14 studies. Additional file 5. Skills covered in the educational intervention identified in this review: summary of the life-and-work skills provided through education programs that were identified in this review. 

Supplementary Material 2.

## Data Availability

All data generated or analysed during this study, including template data collection forms; data extracted from included studies; and data used for all analyses are available at https://github.com/chanyingcrystal/Migrant-worker-health.

## Abbreviations

LEAs: Lived-experience advisors
ES: Effect Size
JBI: Joanna Briggs Institute
MMTA: Mixed Methods Appraisal Tool
NPOs: Non-profit organizations
PRISMA: Systematic Reviews and Meta-Analyses guidelines
CBT: Cognitive-behavioural therapy
CES-D: 8 Measured by the 20-item Centre for Epidemiologic Studies Depression
COPSOQ: Copenhagen Psychosocial Questionnaire
CSI: Caregiver Strain Index
HSCL-25: 12 Measured by the Hopkins Symptom Checklist-25
MBC: Mothers and Babies Course
MFWSI: 13 Measured by the 39-item Migrant Farmworker Stress Inventory
MSDs: Musculoskeletal disorders
PAI: 14 Measured by the 24-item Personality Assessment Inventory
PDSS-SF: 10 Measured by Postpartum Depression Screening Scale, Short-form
QoF: Quality of Life
RWP: Refugee Well-Being Project
SAD: Social Anxiety Disorder
SCL-90: 90-items symptom checklist
SD: Standard Deviation
SEW: Social and Emotional Well-Being
SLA: 6 Measured by Satisfaction with Life Areas scale
WIC: Women Infants and Children
WSI: Work Stressor Inventory
