# Supplementary material for: Educational programmes for reducing depression, anxiety, and stress symptoms among migrant workers: a scoping review and evidence synthesis
Source: BMC Public Health. 2025 Dec 2;26:76. doi: 10.1186/s12889-025-25775-6 (PMC12777341; doi:10.1186/s12889-025-25775-6)
Supplement: Supplementary file 1 — Supplementary Material 1. Additional file 1. List of databases searched and the search strategies used: listed full search term had been used. Additional file 2. Study inclusion criteria: outlined the study selection criteria. Additional file 3. Involvement of lived-experience advisors: detailed account of advisor involvement. Additional file 4. Quality assessment reports: quality assessment reports for all 14 studies. Additional file 5. Skills covered in the educational intervention identified in this review: summary of the life-and-work skills provided through education programs that were identified in this review. [file 12889_2025_25775_MOESM1_ESM.docx]

Additional file 1. List of Databases Searched and the Search Strategies Used

**1.1 OVERVIEW OF THE SEARCH**

Bibliographic databases including Embase via Ovid (1910 - Present), Medline via Ovid (1946 - Present), CINAHL Complete via EBSCOhost (1941 – Present), APA PsycInfo via OVID (1806 – Present), SocINDEX (1895 – Present), and Cochrane Library (1996 – Present), Our search strategies included migrant, education and training, workplace, and stress, anxiety and depression.Manual searches from sources of grey literature including the websites and reports published in the recent 10 years by the International Labour Organization (ILO), the International Organization of Migrant (IOM), the World Health Organization (WHO), the United Nations Refugee Agency (UNHCR), the UN-Habitat, the European Commission, the United Nations Educational, Scientific and Cultural Organization (UNESCO), and the United Nations Development Programme (UNDP) were performed alongside the systematic search. Search engines (Google, Google Scholar and Scopus) were also searched

**1.2 SEARCH STRATEGIES USED**

In this review, we have adopted the below PICO (Population, Intervention, Comparison group, Outcome) statement in formulating the search strategies in different databases.

| **Population (P):** | Migrant workers |
| --- | --- |
| **Intervention (I):** | Education OR empowerment OR life skill |
| **Comparison (C):** | N/A |
| **Outcome (O):** | stress OR anxiety* OR depression* |

1.2.1 Embase via Ovid (1910 - Present)

| **#** | **Search words** | **PICO** |
| --- | --- | --- |
| 1 | migrant.ab,ti. or migrant/ or forced migrant/ or long distance migrant/ or migrant worker/ or short distance migrant/ | P |
| 2 | undocumented immigrant/ or immigrant/ or immigrant.ab,ti. | P |
| 3 | (work* empowerment).ab,ti. or empowerment/ | I |
| 4 | education/ or education.ab,ti. or adult education/ or education program/ or (vocation* education or staff development).ab,ti. | I |
| 5 | workers right*.ab,ti. or workers rights/ or human rights/ or trade union/ | I |
| 6 | workplace.ab,ti. or occupational health/ or workplace/ or occupational safety/ or work environment/ | I |
|  |  |  |
| 7 | "Depression, Anxiety and Stress Scale"/ or Self-rating Anxiety Scale/ or "Depression, Anxiety and Stress Scale-21"/ or anxiety.ab,ti. or anxiety disorder/ or generalized anxiety disorder/ or Generalized Anxiety Disorder-2/ or anxiety/ or Generalized Anxiety Disorder Scale/ or "mixed anxiety and depression"/ or Generalized Anxiety Disorder-7/ or "Depression, Anxiety and Stress Scale-42"/ or anxiety.ab,ti. | O |
| 8 | Edinburgh Postnatal Depression Scale/ or postnatal depression/ or depression/ or central nervous system depression/ or long term depression/ or respiration depression/ or major depression/ or Hamilton Depression Rating Scale/ or agitated depression/ or bipolar depression/ or chronic depression/ or Geriatric Depression Scale/ or atypical depression/ or "mixed mania and depression"/ or depression inventory/ or depression assessment/ or depression.ab,ti. | O |
| 9 | mental stress.ab,ti. or mental stress/ or physiological stress/ | O |
| 10 | 1 or 2 | P |
| 11 | 3 or 4 or 5 | I |
| 12 | 7 or 8 or 9 | O |
| 13 | 10 and 11 and 12 and 6 |  |

1.2.2 Medline (1946-2021)

| **#** | **Search words** | **PICO** |
| --- | --- | --- |
| 1 | migrant.ab,ti. or "Transients and Migrants"/ OR "Emigrants and Immigrants"/ | P |
| 2 | (Empowerment or worker right*).ab,ti. or Empowerment/ or Power, Psychological/ | I |
| 3 | Education.ab,ti. or Competency-Based Education/ or Education/ or adult education/ or education program/ | I |
| 4 | (vocational education or staff development).ab,ti. or Vocational Education/ OR staff development.ab,ti. | I |
| 5 | Workplace/ or (workplace or work environment or occupation*).ab,ti. | I |
| 6 | Anxiety/ or Anxiety.ab,ti. or Anxiety Disorders/ | O |
| 7 | depression.ab,ti. or Depression/ or Long-Term Synaptic Depression/ | O |
| 8 | Stress, Psychological/ or Stress, Physiological/ or stress.ab,ti. OR mental stress.ab,ti. or Occupational Stress.mp | O |
| 9 | 2 OR 3 OR 4 OR 5 | P |
| 10 | 6 OR 7 OR 8 | I |
| 11 | 1 AND 9 AND 10 | O |

1.2.3 CINAHL Complete via EBSCOhost (1941 – Present)

| **#** | **Search words** | **PICO** |
| --- | --- | --- |
| 1 | "Immigrants" OR (MH "Immigrants") OR (MH "Relocation") OR (MH "Emigration and Immigration") OR (MH "Refugees") OR (MH "Transients and Migrants") OR (MH "Undocumented Immigrants") | P |
| 2 | (MH "Empowerment") OR "Empowerment" OR worker right* | I |
| 3 | (MH "Education") OR "education" OR (MH "Vocational Education") OR (MH "Staff Development") OR (MH "Outcomes of Education") or “staff development” | I |
| 4 | (MH "Employee Rights") OR (MH "Legislation, Labor") | I |
| 5 | (MH "Work Environment") OR (MH "Workforce") OR workplace | I |
| 6 | (MH "Depression") OR "depression" OR (MH "Self-Rating Depression Scale") OR (MH "Geriatric Depression Scale") OR (MH "Center for Epidemiological Studies Depression Scale") OR (MH "Beck Depression Inventory, Revised Edition") | O |
| 7 | (MH "Anxiety") OR "anxiety" OR (MH "Social Anxiety Disorders") OR (MH "Anxiety Disorders") OR (MH "Separation Anxiety") OR (MH "Generalized Anxiety Disorder") OR (MH "Anxiety (NANDA)") OR (MH "Anxiety Reduction (Iowa NIC)") OR (MH "Anxiety Control (Iowa NOC)") | O |
| 8 | (MH "Stress") OR "stress" OR (MH "Stress, Occupational") OR (MH "Stress, Physiological") OR (MH "Stress, Psychological") OR (MH "Stress Management") OR (MH "Mental Health") OR "mental stress" | O |
| 9 | 2 OR 3 OR 4 OR 5 | I |
| 10 | 6 OR 7 OR 8 | O |
| 11 | 10 AND 11 AND 12 |  |

1.2.4 APA PsycInfo via OVID (1806 – Present)

| **#** | **Search words** | **PICO** |
| --- | --- | --- |
| 1 | exp Domestic Service Personnel/ or exp Migrant Workers/ or exp Migrant Farm Workers/ or Migrant.ab,ti. or exp Human Migration/ or exp Immigration/ | P |
| 2 | exp Vocational Education/ or (Education OR vocation* education or staff development).ab,ti. or exp Education/ | I |
| 3 | (Empowerment).ab,ti. or exp Empowerment/ | I |
| 4 | exp Community Advocacy/ or Advocacy.ab,ti. or exp Advocacy/ or exp Self-Advocacy/ | I |
| 5 | exp Working Conditions/ or exp Human Rights/ or exp Labor Unions/ or exp Civil Rights/ or worker* right.ab,ti. or exp Activism/ | I |
| 6 | exp Workplace Intervention/ or workplace.ab,ti. or worker.ab,ti. or exp Personnel/ | I |
| 7 | exp Generalized Anxiety Disorder/ or exp Anxiety Management/ or exp Separation Anxiety Disorder/ or exp Speech Anxiety/ or exp Health Anxiety/ or exp Anxiety Disorders/ or exp Anxiety/ or Anxiety.ab,ti. or exp Performance Anxiety/ or exp Social Anxiety/ | O |
| 8 | exp "Long-term Depression (Neuronal)"/ or exp "Depression (Emotion)"/ or exp Major Depression/ or exp Reactive Depression/ or exp Beck Depression Inventory/ or Depression.ab,ti. or exp Endogenous Depression/ or exp Atypical Depression/ or exp Recurrent Depression/ or exp Dysthymic Disorder/ or exp Cyclothymic Disorder/ or exp Internalizing Symptoms/ or exp Bipolar II Disorder/ or exp Affective Disorders/ | O |
| 9 | exp Occupational Stress/ or exp Chronic Stress/ or exp Stress Management/ or exp Stress/ or exp "Stress and Coping Measures"/ or exp Perceived Stress/ or exp Acute Stress Disorder/ or exp Stress Reactions/ or mental stress.ab,ti. or exp Social Stress/ or exp "Stress and Trauma Related Disorders"/ or exp Psychological Stress/ or exp Work Related Illnesses/ | O |
| 10 | 2 OR 3 OR 4 OR 5 OR 6 OR 7 | I |
| 11 | 7 OR 8 OR 9 | O |
| 12 | 1 AND 10 AND 11 |  |

1.2.5 SocINDEX (1895 – Present)

| **#** | **Search words** | **PICO** |
| --- | --- | --- |
| 1 | DE "MIGRANT labor" OR DE "WOMEN foreign workers" OR DE "FOREIGN workers" OR DE "RETURN migrants" OR DE "IMMIGRANTS" | P |
| 2 | DE "EDUCATION" OR DE "WORK & education" OR DE "VOCATIONAL education" OR DE "OCCUPATIONAL training". | I |
| 3 | DE "EMPLOYEE empowerment" or worker* right. | I |
| 4 | DE “WORK environment” or workplace. | I |
| 5 | DE “ANXIETY” OR DE “ANXIETY disorders” OR DE “GENERALIZED anxiety disorder”. | O |
| 6 | DE “MENTAL depression” OR DE “BIPOLAR disorder” OR DE “PSYCHOTIC depression” OR DE “AFFECTIVE disorders”. | O |
| 7 | DE “JOB stress” OR DE “PSYCHOLOGICAL stress” OR DE “ACUTE stress disorder” OR DE “PSYCHOLOGICAL burnout”. | O |
| 8 | 2 or 3 or 4 | I |
| 9 | 5 or 6 or 7 | O |
| 10 | 1 and 8 and 9 |  |

1.2.6 Cochrane Library (1996 – Present)

1. (migrant OR immigrant OR foreign OR refugee) AND (empowerment OR education OR labour right) AND (depression OR anxiety OR stress) AND (workplace)

1.2.7 Scopus, other search engines and organization webpages

1. (migrant OR immigrant OR foreign OR refugee) AND (empowerment OR education OR labour right) AND (depression OR anxiety OR stress) AND (workplace)

**Additional file 2.** **Study inclusion criteria**

| Study design: |
| --- |
| Not exclusive to any of the study designs. |
| Study population: |
| Participants were migrants, regardless of age, gender, race, nationality, or geographical locations, who were working or being employed and for which their psychological conditions were measured and reported. |
| Data: |
| There were extractable data on migrants’ psychological conditions before, during, and/or after joining the educational programmes. If the study included migrants and non-migrants, only data on migrants were extracted. If the study included outcomes beyond our prespecified psychological conditions, only data on anxiety, depression, and stress were extracted. |
| Educational intervention: |
| The topic through which the participant’s psychological conditions were measured and reported was educational intervention. This included:  1.     Work skill education that focused on skills and knowledge required in performing the job (e.g. nursing skills training for healthcare workers);  2.     Life skill education that prepared workers to adapt to the culture and daily-living in host countries (e.g. trainings on languages, job application, and patient education on how to seek healthcare);  3.     Education that involved both work-and-life skills intervention focusing on the general wellbeing in the workers’ daily lives, particularly in workplaces; and psychological intervention approaches (e.g. psychoeducation, spiritual retreat and mental first-aid skills).  Educational interventions should include a description on its structure and intended learning outcomes, and information dissemination (e.g. via educational pamphlet).  Effects of educational intervention on the prevention, management, or stopping occurrence/relapse from stress, depression, or anxiety were sought.  Regular schooling was excluded in this review. |
| Reporting language: |
| Only papers written in English and Chinese were included. |
| Report format: |
| No restriction to the report format. Empirical studies published in peer-reviewed journals and non-peer-reviewed grey literature including reports were included.  Papers without full-text access were not included. |

**Additional file 3. Involvement of Lived-experience Advisors**

In this review, we have engaged lived-experience advisors from seven non-profit organizations (NPOs) serving migrant workers in Hong Kong, Malaysia, Phillipiness, Japan, and Taiwan. We engaged the advisors in idea formulation and data screening through informal communications, where we presented our ideas and invited their immediate feedback to shape the research questions. We also actively invited them to contribute to the pool of literature for our screening and formulate insights on the practical needs of migrant workers. Then, we consolidated the findings of our literature review and invited comments from our advisors through online focus groups of 6 to 9 advisors and supplemented by in-depth interviews through Zoom meetings. Based on their comments in the interviews, we conducted further analyses and incorporated their practical insights into our Discussion section of this article.

**Table A1.** Involvement of lived-experience advisors in different stage of this review

| **Stage** | **Methods** | **Impact on review progress** |
| --- | --- | --- |
| 1. Idea formulation | Informal communications, email conversations, and phone calls to collect feedback | Advise and contribute to the formulation of research questions to be focused on |
| 2. Data collection and study selection |  | Invite advisors to recommend and contribute to the pool of literature for further study selection |
| 3. Analysis and discussion | - Three-hour online focus group discussions with 9 lived-experience advisors who are migrant workers, and experienced leaders in serving the migrant worker community - Two online, semi-structured individual interviews that lasted for 1 and 1.5 hour were conducted with advisors who did not participate in the focus group interview | Collect practical insights from the lived-experience advisors on our consolidated research findings, and therefore to guide direction in further interpretation of the research findings, and writing of report |
| 4. Results disseminations after completion of commission | Informal communications and email conversations | Disseminate study results to important actors in the field |

**Additional file 4. Quality Assessment Reports**

| **RANDOMISED-CONTROLLED TRIAL** | | | | | | | | | | | | | | | |
| --- | --- | --- | --- | --- | --- | --- | --- | --- | --- | --- | --- | --- | --- | --- | --- |
| Study | Appraisal tool | Items | | | | | | | | | | | | | Sum of score |
|  |  | Q1: Randomisation | Q2: Concealed intervention allocation | Q3: Similar intervention groups at baseline | Q4: Blinding to intervention assignment | Q5: Blinding to intervention providers | Q6: Blinding to outcome assessors | Q7: Same treatment other than intervention of interest | Q8: Follow-up information | Q9: Intention-to-treat analysis | Q10: Same outcome measurement for intervention group | Q11: Reliable outcome measured | Q12: Appropriate statistical analysis | Q13: Appropriate trial design |  |
| Kocken (2008) | JBI RCT Checklist | Yes | No | Yes | No | No | No | Yes | Yes | No | Yes | Yes | Yes | Yes | 8 out of 13 |

| Study | Appraisal tool | Domain 1 | | | | Domain 2 | | | | | | | | Domain 3 | | | | | Domain 4 | | | | | | Domain 5 | | | | Overall Bias |
| --- | --- | --- | --- | --- | --- | --- | --- | --- | --- | --- | --- | --- | --- | --- | --- | --- | --- | --- | --- | --- | --- | --- | --- | --- | --- | --- | --- | --- | --- |
|  |  | 1.1 | 1.2 | 1.3 | 1.0 result | 2.1 | 2.2 | 2.3 | 2.4 | 2.5 | 2.6 | 2.7 | 2.0 result | 3.1 | 3.2 | 3.3 | 3.4 | 3.0 result | 4.1 | 4.2 | 4.3 | 4.4 | 4.5 | 4.0 result | 5.1 | 5.2 | 5.3 | 5.0 result |  |
| Kocken (2008) | Cochrane RoB 2 | Y | Y | N | Low | Y | Y | N | NA | NA | Y | NA | Low | PY | NA | NA | NA | Low | N | N | NI | N | NA | Low | Y | N | N | Low | Low |

| **COHORT STUDY** | | | | | | | | | | | | | |
| --- | --- | --- | --- | --- | --- | --- | --- | --- | --- | --- | --- | --- | --- |
| Study | Appraisal tool | Items | | | | | | | | | | | Sum of score |
|  |  | Q1: Recruitment | Q2: Exposure measurement | Q3: Reliable exposure measured | Q4: Confounding | Q5: Resolution of confounding | Q6: Outcome-free exposure | Q7: Reliable outcome measured | Q8: Follow-up time | Q9: Reason to loss to follow-up | Q10: Address incomplete loss to follow-up | Q11: Appropriate statistical analysis |  |
| Goodkind (2005) | JBI Cohort Study Checklist | NA | NA | NA | No | No | Unclear | Yes | Yes | Yes | Yes | Yes | 5 out of 8 |
| Hovey (2014) |  | NA | NA | NA | No | NA | No | Yes | Yes | No | No | Yes | 3 out of 7 |
| Poudel-Tandukar (2021) |  | NA | NA | NA | No | NA | Yes | Yes | No | Yes | NA | Yes | 4 out of 6 |

| **QUASI-EXPERIMENTAL STUDY / NON-RANDOMISED EXPERIMENTAL STUDY** | | | | | | | | | | | |
| --- | --- | --- | --- | --- | --- | --- | --- | --- | --- | --- | --- |
| Study | Appraisal tool | Items | | | | | | | | | Sum of score |
|  |  | Q1: Clear cause & effect | Q2: Similar participants in comparisons | Q3: Good control of environment | Q4: Control group | Q5: Multiple outcome measurement | Q6: Follow-up information | Q7: Same outcome measurement in comparisons | Q8: Reliable outcome measured | Q9: Appropriate statistical analysis |  |
| Wu (2005) | JBI Quasi-experimental study Checklist | Yes | Yes | Yes | Yes | Yes | Yes | Yes | Yes | Yes | 9 out of 9 |
| Tran (2014) |  | Yes | Yes | Yes | No | Yes | No | NA | Yes | Yes | 6 out of 8 |
| Ekwonye (2018) |  | Yes | Yes | Yes | No | Yes | Yes | NA | Yes | Yes | 7 out of 8 |

| **CROSS-SECTIONAL STUDY** | | | | | | | | | | |
| --- | --- | --- | --- | --- | --- | --- | --- | --- | --- | --- |
| Study | Appraisal tool | Items | | | | | | | | Sum of score |
|  |  | Q1: Clear inclusion criteria | Q2: Detailed settings | Q3: Reliable exposure measured | Q4: Objective measurement of condition | Q5: Confounding | Q6: Resolution of confounding | Q7: Reliable outcome measured | Q8: Appropriate statistical analysis |  |
| Chuang (2018) | JBI Analytical cross-sectional study checklist | Yes | Yes | NA | Yes | No | No | Yes | Yes | 5 out of 7 |

| **QUALITATIVE STUDY** | | | | | | | | | | | | |
| --- | --- | --- | --- | --- | --- | --- | --- | --- | --- | --- | --- | --- |
| Study | Appraisal tool | Items | | | | | | | | | | Sum of score |
|  |  | Q1: Congruity between the theory & methodology | Q2: Congruity between methodology & research questions | Q3: Congruity between methodology & data collection | Q4: Congruity between methodology & data analysis | Q5: Congruity between methodology & results | Q6: Influence from researchers’ belief | Q7: Address influence of researchers | Q8: Adequate participants’ voices | Q9: Ethical approval | Q10: Conclusion drawn based on data collected |  |
| Mitschke (2017) | JBI Qualitative research checklist | No | Yes | Yes | Yes | Yes | No | No | Yes | No | Yes | 6 out of 10 |

| **CASE SERIES** | | | | | | | | | | | | |
| --- | --- | --- | --- | --- | --- | --- | --- | --- | --- | --- | --- | --- |
| Study | Appraisal tool | Items | | | | | | | | | | Sum of score |
|  |  | Q1: Clear inclusion criteria | Q2: Reliable condition measured | Q3: Valid condition identification | Q4: Consecutive participants inclusion | Q5: Complete participants inclusion | Q6: Clear participants demographics | Q7: Clear clinical information | Q8: Clear outcomes | Q9: Clear sites information | Q10: Appropriate statistical analysis |  |
| Weiss (2011) | JBI Case series checklist | No | No | Yes | No | No | Yes | Yes | Yes | Yes | NA | 5 out of 9 |

| **MIXED-METHODS STUDY** | | | | | | | | | | |
| --- | --- | --- | --- | --- | --- | --- | --- | --- | --- | --- |
| Study | Appraisal tool | Items | | | | | | | | |
|  |  | Screening 1: Clear research question(s) | Screening 2: Data collected addresses research question(s) | Q1.1: Appropriate qualitative approach | Q1.2: Adequate qualitative data collection methods | Q1.3: Findings derived adequately from data | Q1.4: Results sufficiently substantiated by data | Q1.5: Coherence between data source, collection, analysis & interpretation | Q3.1: Representativeness of participants | Q3.2: Appropriate outcome and intervention measurement |
| Goodkind (2014) | MMAT (Qualitative + Quantitative non- | Yes | Yes | Yes | Yes | Yes | Yes | Yes | No | Yes |
| Busch (2017) |  | Yes | Yes | Yes | Yes | Yes | Unclear | Yes | No | Yes |
| Le (2021) |  | Yes | Yes | Yes | Yes | Yes | Yes | Yes | Yes | Yes |

| **MIXED-METHODS STUDY (CONT’)** | | | | | | | | | | |
| --- | --- | --- | --- | --- | --- | --- | --- | --- | --- | --- |
|  |  | Items | | | | | | | | Sum of score |
|  |  | Q3.3: Complete outcome data | Q3.4: Accounted for confounder(s) | Q3.5: Intended intervention administration | Q5.1: Adequate rationale for mixed-methods study | Q5.2: Effective integration of components | Q5.3: Adequate quantitative and qualitative outcome interpretation | Q5.4: Adequately addressed inconsistencies between quantitative and qualitative results | Q5.5: Adherence to quality criteria of each of quantitative or qualitative study |  |
| Goodkind (2014) | randomised + Mixed methods) | Unclear | No | Yes | Yes | Yes | Yes | Yes | Yes | 12 out of 14 |
| Busch (2017) |  | No | Yes | Yes | No | Yes | Yes | Yes | Yes | 11 out of 14 |
| Le (2021) |  | Unclear | No | Yes | Yes | Yes | Yes | Yes | Yes | 13 out of 14 |

| **MIXED-METHODS STUDY (CONT’)** | | | | | | | | | | |
| --- | --- | --- | --- | --- | --- | --- | --- | --- | --- | --- |
|  |  | Items | | | | | | | | |
|  |  | Screening 1: Clear research question(s) | Screening 2: Data collected addresses research question(s) | Q1.1: Appropriate qualitative approach | Q1.2: Adequate qualitative data collection methods | Q1.3: Findings derived adequately from data | Q1.4: Results sufficiently substantiated by data | Q1.5: Coherence between data source, collection, analysis & interpretation | Q4.1: Relevant sampling strategy | Q4.2: Representativeness of sample |
| Smith (2013) | MMAT (Qualitative + Quantitative | Yes | Yes | Yes | Yes | Yes | Yes | Yes | Yes | No |

| **MIXED-METHODS STUDY (CONT’)** | | | | | | | | | | |
| --- | --- | --- | --- | --- | --- | --- | --- | --- | --- | --- |
|  |  | Items | | | | | | | | Sum of score |
|  |  | Q4.3: Appropriate measurements | Q4.4: Low risk of nonresponse bias | Q4.5: Appropriate statistical analysis | Q5.1: Adequate rationale for mixed-methods study | Q5.2: Effective integration of components | Q5.3: Adequate quantitative and qualitative outcome interpretation | Q5.4: Adequately addressed inconsistencies between quantitative and qualitative results | Q5.5: Adherence to quality criteria of each of quantitative or qualitative study |  |
| Smith (2013) | descriptive + Mixed methods) | Yes | No | Yes | Yes | Yes | Yes | Yes | Yes | 13 out of 15 |

**Additional file 5. Skills covered in the educational intervention identified in this review**

| **Skills involved** | **Education recipient** | **Included articles** |
| --- | --- | --- |
| ***Work skills*** |  |  |
| Emotional and instrumental support skills to support workers/ colleagues | Manager | Busch (2017) |
|  | Workers | Busch (2017) |
| Skills to hold meetings effectively | Manager | Busch (2017) |
| Skills to incorporate worker support elements in daily management | Manager | Busch (2017) |
| Advocacy skills in improving work environment in a participative way | Workers | Busch (2017) |
|  | Workers | Mitschke (2017) |
| Vocational training (e.g. nursing care for newly migrated nurse) | Workers | Wu (2005) |
|  | Workers | Chuang (2018) |
|  | Workers | Smith (2013) |
| Job application/ employment-related skills training | Workers | Mitschke (2017) |
|  | Workers | Smith (2013) |
| ***Life skills*** |  |  |
| Host country language learning | Workers | Mitschke (2017) |
|  | Workers | Smith (2013) |
| Stress management skills (e.g mood regulation etc.) | Workers | Ekwonye (2018) |
|  | Workers | Tran (2014) |
|  | Workers | Mitschke (2017) |
|  | Workers | Kocken (2008) |
|  | Workers | Wu (2005) |
|  | Workers | Le (2022) |
|  | Workers | Weiss (2011) |
| Skill to access to resource in host countries (e.g health care, housing, prepare citizenship exam, communication skills with doctors) | Workers | Tran (2014) |
|  | Workers | Mitschke (2017) |
|  | Workers | Kocken (2008) |
| Social networking skills and peer knowledge exchange | Workers | Tran (2014) |
|  | Workers | Mitschke (2017) |
|  | Workers | Wu (2005) |
| Health education (understanding mental illness, including the symptoms, risk factors etc.) | Workers | Kocken (2008) |
|  | Workers | Tran (2014) |
| Skills in relation to parenting and handling family conflict | Workers | Hovey (2014) |

**Additional file 6.**  Practical Insights from Lived-experience Advisors

We engaged our advisors since the idea generation and methodology formulation phase of our review. As our advisors noticed that detailed documentation of community intervention conducted by NPOs was scarce, we decided to actively invite them to provide relevant reports for our review and selection process, so as to present a wider coverage of existing literatures on migrant workers’ health. In addition, we conducted online focus groups, supplemented by individual semi-structured interviews for advisors who could not join the group discussion, so they could therefore communicate their lived experiences in the field and help guide further analysis and insight.

In both focus groups and individual interviews, the advisors recognized the interventions we included were relevant to mental health promotion of migrant workers during acculturation, especially culturally-sensitive approaches such as spiritual-training, lay health educator, and daily-living training through migrant-local support groups. They shared that work-and-life skills education programs were important to workers’ mental wellbeing, as “they were skills that we used everyday but no one would ever teach us”.  In terms of specific interventions, the advisors shared that a lay health educator approach was particularly helpful in motivating behavioural change in new migrants, to whom help from peers was more acceptable. Advisors also shared that culturally-sensible interventions that were incorporated with life skill training – for example, spiritual training interventions – were easier to adopt in some ethnic groups such as the Filipinos and Malaysians. The advisors also highlighted education on mental health literacy, and particularly suicidal management, was very important. More than one advisor mentioned that they had encountered migrant workers attempting suicide in the host countries; and work-and-skills education on mental health literacy was essential for managing such critical situations.

The advisors also identified gaps in implementing work-and-life skills interventions. While most of the community engagements depended on the leadership of the labour’s unions and NPOs, lack of sustainable manpower and financial support might limit the capacity for NPOs to implement additional services, including the work-and-life intervention highlighted in this review. The unions’ legal rights and accountabilities were deemed to be important for implementation. However, as shared by our advisors, in Malaysia and Kuwait, migrant workers are not allowed to establish labour unions. As such, they can only join existing unions as ordinary members, but cannot be elected to union leadership roles. Policies supporting the development of NPOs are crucial in this landscape; while legal enforcement on mechanisms that ensure employees’ participation in business corporate governance remains scarce in Asia.

The advisors also noted that culturally sensible and affordable professional support remains scarce, which limits the feasibility and sustainability of education interventions, as they require professional support such as from clinical psychologists in psychoeducation programmes. Other than the education model reviewed, the advisors highlighted that anti-stigmatization of migrant workers and migrant worker friendly campaigns, should be implemented in parallel with the education sessions, which could facilitate the acculturation of migrant workers in their new host countries and improve their mental health wellbeing.
